# A prospective, randomised, controlled, double blinded, cross-over study on the effect of a single session of pulsed electromagnetic field therapy on signs of hip osteoarthritis in dogs

**DOI:** 10.1186/s13028-024-00754-w

**Published:** 2024-07-26

**Authors:** Gillian Leung, Jouni Junnila, Thomas Björkenheim, Helena Tiainen, Heli Katariina Hyytiäinen

**Affiliations:** 1 School of Veterinary Science, University of Liverpool, Leahurst Campus, Chester High Road, Neston, CH64 7TE UK; 2 EstiMates Oy, Kamreerintie 8, Espoo, 02770 Finland; 3 Department of Equine and Small Animal Medicine, Faculty of Veterinary Medicine, University of Helsinki, P.O.Box 57, 00014 Helsinki, Finland; 4 Veterinary Teaching Hospital, University of Helsinki, P.O.Box 57, 00014 Helsinki, Finland

**Keywords:** Canine, Degenerative joint disease, Lameness, PEMFT, Physiotherapy, Rehabilitation

## Abstract

**Background:**

Canine coxofemoral joint osteoarthritis is a common, painful and debilitating condition. The objective of this study was to evaluate if any measurable changes in pain or lameness occurred in this patient group immediately after a single treatment with pulsed electromagnetic field therapy. Eight dogs with coxofemoral joint osteoarthritis presenting with signs of pain and lameness were prospectively recruited to this randomised, controlled, double blinded, cross-over study. Subjects attended the research facility on two occasions for one active and one placebo treatment with pulsed electromagnetic field therapy. The immediate effect of one pulsed electromagnetic field therapy treatment on pain and lameness was measured subjectively with the Helsinki Chronic Pain Index and Visual Analogue Scale and objectively using a pressure sensitive walkway.

**Results:**

A statistically significant difference (*P* = 0.03) for change in stride length in the affected limb was recorded for subjects between the active and placebo treatments with pulsed electromagnetic field therapy. Within the active treatment results, there was a statistically significant change in the measurement for reach (*P* = 0.04) and stride length (*P* = 0.047) which got shorter in the affected limb post treatment. For the subjective outcome measures, there was no statistically significant difference between the active and placebo treatments for the evening of the treatment day or the next morning from pre-treatment values. Within the placebo treatment results a statistically significant change (improvement) was detected in Visual Analogue Score (*P* = 0.03) between pre-treatment and the next morning values.

**Conclusions:**

The findings of this study do not show demonstrable improvement in owner assessed pain levels or temporospatial performance in dogs with coxofemoral joint osteoarthritis immediately after a single application of pulsed electromagnetic field therapy.

## Background

Canine osteoarthritis (OA) is a painful and debilitating condition estimated to affect up to 38% of dogs in the United Kingdom [1]. One of the most common joints affected is the coxofemoral (hip) joint [2]. This progressive disease can lead to persistent clinical signs of pain and lameness as the dog attempts to redistribute their weight more comfortably [3]. Owners may notice changes such as more effort to get up from a lying position, shortened steps, decreased swing phase flight arch, difficulty climbing stairs or reluctance to go out [4]. This affects the dog’s functionality and quality of life, causes worry for the owner and can be expensive to treat [5]. Owners become highly aware of their dog’s pain and functionality meaning that validated subjective questionnaires, such as the Helsinki Chronic Pain Index (HCPI) and visual analogue scale (VAS), are often used to measure severity of OA signs [6, 7]. A multimodal management approach within veterinary medicine for this patient group currently includes pharmaceuticals (including analgesics and anti-inflammatory medication), environment and lifestyle modification, weight management, surgical intervention, acupuncture, physiotherapy and hydrotherapy [8–12]. A knowledge gap exists in the understanding of the role that these treatments play in the successful management of OA. Thus, there is a need for further evidence based, affordable, effective and non-invasive treatments for this patient group. The clinical impact of such a pain management method would directly affect those dogs who suffer from hip OA, as well as their owners.

Pulsed electromagnetic field therapy (PEMFT) is the application of low frequency, athermal, electromagnetic fields by running an electric current through a coiled wire to produce therapeutic effects, based on Faraday’s Law of induction [13, 14]. Electromotive forces induced in the cell membranes of connective tissues result in various physiological effects ultimately serving to control pain or enhance bone or tissue healing [15]. Specific effects include reduced levels of nitric oxide and mitochondrial free radical production, increased blood and lymph flow which reduces pain and oedema [16–20]. Studies examining the effect of PEMFT on inflammatory mediators in synovitis and traumatic brain injury have shown increased phagocytosis of neutrophils by macrophages and reduced prostaglandins and inflammatory cytokines by activation of adenosine receptors in the cell membrane [21–23]. Of particular relevance to OA management are in-vitro studies using rats showing inhibition of cartilage degeneration and preservation of subchondral bone integrity with PEMFT [24, 25].

Systematic reviews in human medicine have determined PEMFT to be an effective treatment for alleviating symptoms of pain, stiffness and functional impairment caused by OA [26–28]. Conversely, one systematic review suggested PEMFT is no more effective than other conservative modalities such as heat or shock wave therapy at improving osteoarthritic symptoms in humans [29]. Several studies demonstrate positive effects with PEMFT for dogs in, for example, pain after hemilaminectomy, wound healing and Legg-Calve-Perthes disease [30–33]. Conversely, no statistically significant effect was shown using PEMFT for treatment of equine back pain [34]. Two studies specifically investigating the effects of PEMFT on dogs with OA concluded it is a potentially beneficial treatment modality for relieving pain and improving functional ability [31, 35]. Both these studies, however, have methodological flaws including lack of owner blinding. There is a lack of prospective, blinded, randomised controlled trials (RCTs) regarding the use of PEMFT in the existing evidence base [36]. Specifically, there is a lack of evidence to guide clinicians using this modality as to which treatment parameters are most beneficial. Current clinical observations of therapists’ and owners’ expectations of immediate effects after single PEMFT treatment influenced our study design. The objective of this study was to determine if one single session of PEMFT has an immediate effect on temporospatial variables of gait and owner assessed pain in dogs with hip OA. The hypothesis was that PEMFT would improve temporospatial variables of the treated limb immediately after treatment. The second hypothesis was that owner assessed pain levels of the dogs would decrease in the 24 h after PEMFT.

## Methods

### Animals

Privately owned pet dogs were recruited via advertisement on the University of Helsinki Veterinary Teaching Hospital’s Facebook page with participating owners signing informed consent. Inclusion criteria were dogs weighing 10–60 kg aged between two and ten years old with hip OA diagnosed by a veterinarian. The dogs were to display signs of pain, lameness or activity limitations (such as difficulty with stairs) despite potentially taking OA related medication such as non-steroidal anti-inflammatories (NSAIDs). Any existing individual drug therapy would be maintained for subjects during the study. Diagnosis was to have been made at least one month prior to study participation, with no changes in medication within the preceding two weeks. Both unilaterally and bilaterally affected dogs were included. The hip showing most clinical signs (i.e., pain, muscle atrophy, lameness or reduced range of motion) at the time of participation in the study was determined by the orthopaedic specialist vet’s assessment as the one to be treated. Exclusion criteria comprised presence of pacemaker or metallic implants, neurological or endocrine disease, cardiac conditions, malignancy, pregnancy, infections and brachycephalia, or clinical findings contributing to abnormal movement in the spine or other joints apart from the hip joint during the orthopaedic examination at the start of the study. All pre-existing pain medication continued during the study period. If medication changes were necessary during the study period, the subject was withdrawn. No other physiotherapy intervention was permitted during data collection or washout period. The subjects daily exercise and activities continued as normal. The study was approved by the University of Helsinki, Campus Ethical Review Board (approval number 12/2020) and the University of Liverpool Ethics Committee (approval number VREC1046).

### Study design

**Fig. 1:**
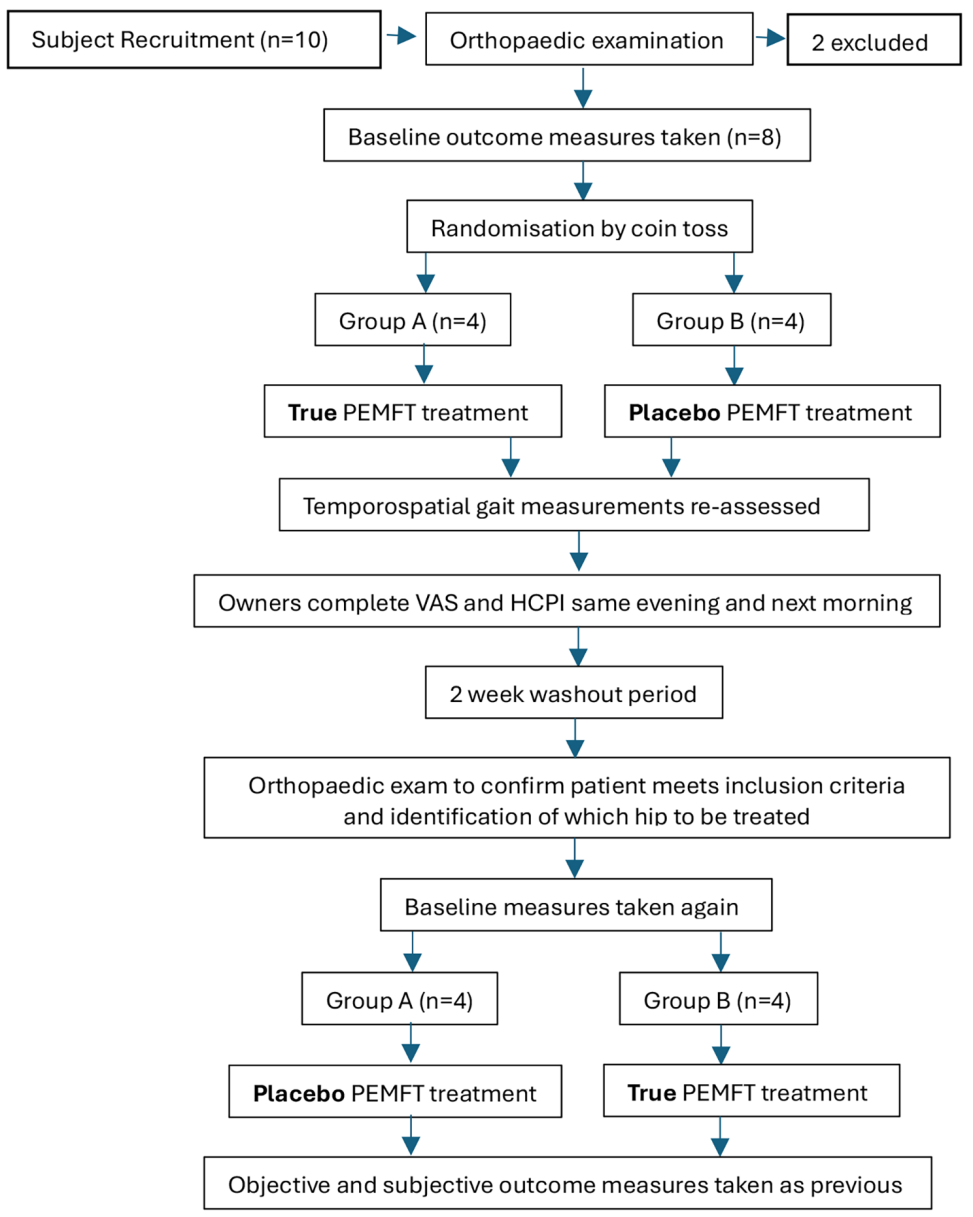
Illustration of study design

The study design was a prospective, randomised, controlled, double blinded, cross-over study. It was conducted at the Veterinary Teaching Hospital, University of Helsinki, Finland, between February and March 2022. Sample size calculation was conducted using ‘Epitools’ software (Sergeant, ESG, 2018. Epitools Epidemiological Calculators. Ausvet). Using a paired t-test, assuming an effect size of 0.5 and standard deviation of 0.49 of the change in the outcome (based on questionnaire results in a previous study [31]) eight dogs were required to achieve 80% power with a type 1 error rate of 0.05. To allow for dropouts, 10 dogs were recruited. The dogs were divided into two sequences, and they attended the physiotherapy department at The University of Helsinki for one true (active) PEMFT treatment and one placebo PEMFT treatment spaced 14 days apart which served as a standardised washout period (Fig. 1). Thus, each dog served as their own control.

At the start of each treatment session, firstly, a standardised clinical, orthopaedic examination was performed by a specialist orthopaedic veterinarian (one of the authors, TB) to exclude any clinical findings other than signs of hip OA [37]. The examination comprised visual evaluation of the animal (including body condition scoring on a scale of 1–5 where 3 is normal), lameness assessment on a scale from 0 to 4 (where 0 = no lameness and 4 = non-weightbearing lameness), palpatory assessment of limbs and spine evaluating pain, swelling, crepitations and range of motion. Examination of the hip joints was done by mild flexion, extension, abduction, rotation and palpation to decide which hip was worse and more painful. Testing of soft tissue structures including collateral ligaments, medial compartment test for the elbow and tibial thrust for the stifle were also performed.

Objective measurements (performed by one of the authors, HKH) using a pressure sensitive walkway (GAITRite Electronic Walkway, Peekskill, NY, USA) comprised temporospatial values of trot: reach (cm), total pressure (TPI), stance time (secs), step length (cm) and stride length (cm). Subjects were acclimatised to the environment and equipment prior to measurements being recorded by allowing them to walk around the room and on the pressure sensitive walkway for ten minutes. The dogs were then trotted over the walkway, led by their owners. To ensure the dog’s natural movement was unaffected, owners were instructed to keep their dog on a loose lead and use no treats or eye contact. Measurements were taken in both directions, to ensure equal side effect of the owner on the results [38]. In total, a minimum of 10 full gait cycles were recorded for each dog on both visits. The measurements were recorded after the dogs’ orthopaedic examination prior to PEMFT treatment and repeated immediately afterwards. Owners were asked to complete the validated HCPI questionnaire and VAS addressing the perceived pain level of their dog prior to the treatment, in the evening of the treatment day at home and the following morning [6, 7]. This process was performed for the subjects in the same way on both visits.

**Fig. 2:**
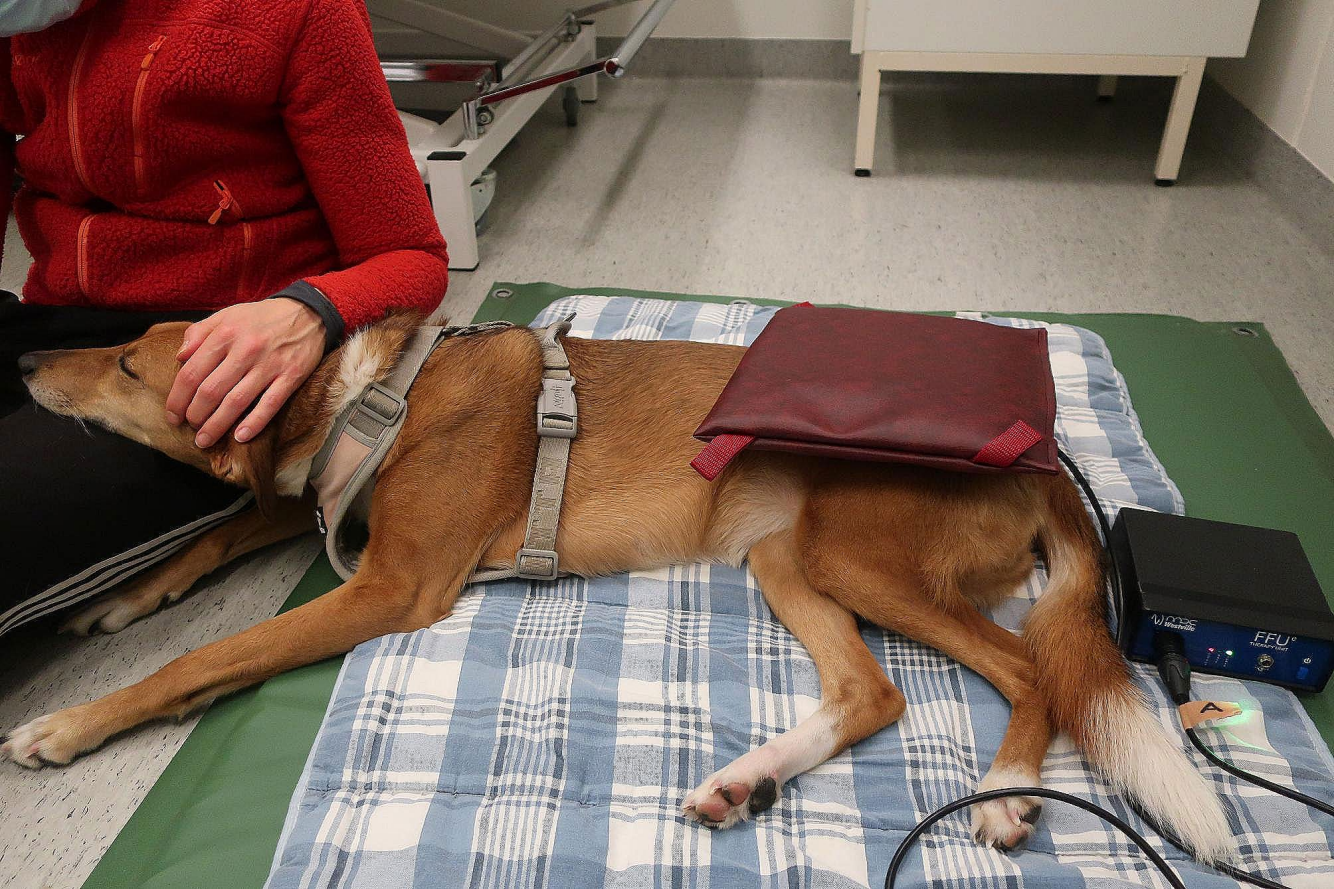
Experimental set up for application of PEMFT treatment showing device used and position of subjects

Subjects received PEMFT (Westville Therapy Fixed Frequency Unit, Westville Therapy Systems, England) on both occasions with one application being active treatment and one being placebo treatment. Randomisation by coin toss determined the order of active or placebo treatment. The treatment protocol was applied by the same therapist (one of the authors, HT) at each session with the placebo treatment using a disabled treatment pad. The placebo treatment pad was disabled by an internal wire of the treatment pad being disconnected meaning that although the base unit would still light up, no electric charge entered the pad. Double blinding for both researchers and owners was ensured by having no external clues as to which pad was in use. Subjects were positioned in lateral recumbency on a large, padded, foam mat with the most symptomatic hip uppermost. The PEMFT treatment coil was positioned on the uppermost hip with the greater trochanter in the centre of the coil (Fig. 2). The treatment parameters of the machine were set at a base frequency of 200 Hz with constant pulse as per manufacturers guidance and based upon early, in vitro work [39]. Treatment time was 15 min with the owner present throughout. At the end of the data collection period, the therapist who applied the treatment was asked to identify which treatment pad was active and which was placebo based on their observations of the subjects behaviour or reaction during treatment.

### Data analysis

Descriptive statistics were calculated for the background variables (age, weight, gender, breed and use of medication) for the whole study population. Descriptive statistics for walkway measurements (reach, total pressure, stance time, step length and stride length) are presented in Table 1. Pain variables (HCPI, VAS) were calculated by treatment and time point. Changes from pre-treatment were also calculated. Changes from pre-treatment in walkway measurements were analysed with linear mixed effects models suitable for the 2 × 2 cross-over design. Treatment period and treatment sequence were used as fixed terms and dog was used as a random effect in the models. Similar methodology was used to analyse HCPI – total score, separately for the evening and the next morning responses. All statistical calculations were performed using the SAS software version 9.4 (SAS Institute Inc., Cary, NC, US). *P*-values < 0.05 were considered statistically significant.

## Results

### Animals

Initially ten dogs were recruited but two were excluded immediately due to pain being found in joints other than the hip, during the orthopaedic examination. Thus, eight dogs (five female, three male) met the inclusion criteria. Represented breeds were three crossbreeds, and one each of Gordon Setter, Irish Setter, Cocker Spaniel, Belgian Shepherd Dog, and German Shepherd Dog. The dogs’ mean and standard deviation (SD) age was 4.9 (± 2.7) years and body weight was 25 (± 8) kg. Only two dogs were on medication for OA, which in both cases was a combination of NSAIDs and Cartrophen.

### Orthopaedic examination

At the initial veterinary examination, seven dogs had a normal body condition score of 3 out of 5, and one was overweight (4 out of 5). By the second veterinary examination, one dog had moved into the overweight category. Pain was identified in the symptomatic hip joint for all dogs with six dogs also presenting with crepitations and /or reduced range of motion of the hip joint. Six dogs also had pain in the contralateral hip joint along with reduced range of motion for two dogs and reduced range of motion and crepitus for one dog. Visual examination of lameness grading was mild for four dogs and moderate for four dogs. Three dogs were noted to have mild tenderness in one other area apart from the hip joints (elbow, lumbar spine and stifle) but not to the extent that they would have met the exclusion criteria. Four dogs presented with asymmetrical muscle bulk with the smaller thigh circumference in all cases being on the side of the most symptomatic hip joint. One dog had hip joint laxity during abduction and rotation of the hip joint and one dog was too uncomfortable to allow the test to be done.

### Temporospatial gait analysis

The temporospatial gait analysis measurements were taken before and after treatment for both the active and placebo PEMFT treatments for the study cohort (Table 1). There was a statistically significant difference between the active and placebo treatments for the change in stride length (*P* = 0.03) (Table 2). However, there were no statistically significant differences between the active and placebo treatments for reach, total pressure, stance time or step length between pre and post treatment measurements (*P* > 0.05) (Table 2). When the change was assessed within treatment, the active treatment showed a statistically significant change from pre-treatment values in the measurements for reach (*P* = 0.04) and stride length (*P* = 0.047) (Table 3). The mean change in stride length (*P* = 0.05) from pre-treatment values for the active treatment was 6.03 centimetres. However, there was no statistically significant change in any of the kinematic variables from pre-treatment values for the placebo treatment (*P* > 0.05). The results for reach shown in Table 3 indicate a decrease in this measurement for both active and placebo treatments (shown by the minus sign value).

**Table 1:** Summary of temporospatial gait analysis measurements in the active and placebo treatment cohorts

Kinematic Variable	Pre-treatment (mean+/-SD)	Post-Treatment (mean +/- SD)
**Reach (cm)**		
Placebo treatment	4.81+/- 5.97	4.06 +/- 6.88
Active treatment	6.90 +/- 7.67	4.79 +/-7.45
**Total Pressure (TPI)**		
Placebo treatment	23.77 +/- 8.19	23.30 +/-7.56
Active treatment	24.28 +/- 8.88	24.16 +/- 9.0
**Stance Time (secs)**		
Placebo treatment	0.16 +/- 0.04	0.16 +/- 0.03
Active treatment	0.19 +/-0.08	0.16 +/- 0.03
**Step Length (cm)**		
Placebo treatment	58.34 +/- 10.09	59.61 +/- 7.51
Active treatment	62.42 +/- 12.68	57.58 +/- 11.15
**Stride Length (cm)**		
Placebo treatment	116.34 +/- 19.58	115.58 +/- 19.16
Active treatment	122.86 +/- 23.21	116.83 +/- 22.80

Descriptive statistics of the temporospatial gait analysis measurements – mean values +/- Standard Deviation are presented. Units – cm – centimetres, TPI – Total Pressure Index, secs – seconds

**Table 2:** Difference in change in temporospatial variables between active and placebo treatments, before and after treatment

Kinematic Variable	Estimate	95% Confidence Interval	*P*
**Reach**	-1.36	[-3.62 ; 0.89]	0.19
**Total Pressure**	0.35	[-0.71 ; 1.42]	0.45
**Stance Time**	-0.03	[-0.13 ; 0.06]	0.41
**Step Length**	-6.11	[-12.55 ; 0.34]	0.06
**Stride Length**	-5.27	[-9.63 ; -0.91]	0.03*

Analysis of difference between the active and placebo treatments in change from before-measurement is presented with the estimate of difference (active – placebo), lower and upper values of the confidence interval set at 95% and significance set as *P* < 0.05 marked with *. (*P* = level of significance)

**Table 3:** Change in temporospatial variables within active and placebo treatments, before and after treatment

Kinematic Variable	Estimate	95% Confidence Interval	*P*
**Reach**			
Placebo treatment	-0.75	[-2.76 ; 1.26]	0.41
Active treatment	-2.11	[-2.11 ; -4.13]	0.04*
**Total Pressure**			
Placebo treatment	-0.47	[-1.58 ; 0.64]	0.36
Active treatment	-0.11	[-1.22 ; 0.99]	0.82
**Stance Time**			
Placebo treatment	0	[-0.06 ; 0.06]	0.97
Active treatment	-0.03	[-0.01 ; 0.03]	0.26
**Step Length**			
Placebo treatment	1.27	[-4.42 ; 6.97]	0.61
Active treatment	-4.83	[-10.53 ; 0.86]	0.08
**Stride Length**			
Placebo treatment	-0.76	[-6.7 ; 5.18]	0.77
Active treatment	-6.03	[-11.97 ; -0.09]	0.047*

Analysis of change within active and placebo treatments with pulsed electromagnetic field therapy is presented with the estimate of change from before treatment, lower and upper values of the confidence interval set at 95% and significance set as *P* < 0.05 marked with *. (*P* = level of significance)

### Owner reported pain

A full dataset was not available for either the HCPI or VAS measurements. For the HCPI results, one subject did not have post treatment HCPI results returned for either the evening or next morning time points. For the VAS results one post treatment score for two subjects and two post treatment scores for one of the two subjects were not returned. The other available measurement results for these subjects were included in the analysis.

The mean pre-treatment HCPI was 9.75 ± 5.65 for the active treatment and 8.19 ± 4.80 for the placebo treatment out of a total score of 44. The mean VAS score, which is scored out of 10, for the active treatment was 2.5 ± 1.8 and for the placebo treatment was 1.96 ± 1.19. There was no statistically significant difference (*P* > 0.05) in the mean scores of the HCPI or VAS between the active and placebo treatments for the evening of the treatment day or the next morning from pre-treatment values (Table 4). There was also no statistically significant change in HCPI scores within the active treatment session from pre-treatment values for either of the post treatment time points (Table 5). Within the placebo treatment results, a statistically significant change (improvement) was detected in VAS score (*P* = 0.03) between pre-treatment and the next morning values (Table 5). Sequence effect (dogs reach better results when treatments are received in a certain order) was statistically significant (*P* = 0.02) in the change from pre-treatment VAS score and the evening of the treatment day measurement (dogs receiving PEMFT treatment first reaching better results than dogs receiving placebo first).

When, at the end of data collection, the clinician applying the treatment (blinded in regard to active and non-active treatment pad; i.e., active and placebo), was asked if they could identify which pad was which, they confidently identified them correctly. They made their judgement according to the animal’s reaction during the treatment.

**Table 4:** Change in HCPI and VAS scores *between* active and placebo treatments, before and after treatment

	Estimate	95% Confidence Interval	*P*
**HCPI**			
Evening scores	-0.73	[-3.27 ; 1.80]	0.50
Next morning scores	1.75	[-1.16 ; 4.66]	0.17
**VAS**			
Evening scores	-0.30	[-1.61 ; 1.01]	0.60
Next morning scores	-0.41	[-2.29 ; 1.47]	0.61

Analysis of difference between active and placebo treatment results in change from before-measurement for the owner completed ‘Helsinki chronic pain index (HCPI)’ and ‘visual analogue scale (VAS)’ is presented with the estimate of difference (placebo – active), lower and upper values of the confidence interval set at 95% and significance set as *P* < 0.05 marked with *. (*P* = level of significance)

**Table 5:** Change in HCPI and VAS scores *within* active and placebo treatments, before and after treatment

	Estimate	95% Confidence Interval	*P*
**HCPI Scores (Evening of intervention)**			
Placebo treatment	-0.94	[-3.94; 1.62]	0.40
Active treatment	--0.21	[-1.40; 0.98]	0.97
**HCPI Scores (Morning after intervention)**			
Placebo treatment	-0.19	[-2.16; 1.78]	0.82
Active treatment	-1.94	[-0.82; 0.16]	0.06
**VAS Scores (Evening of interventions)**			
Placebo treatment	-0.65	[-1.36; 0.06]	0.07
Active treatment	-0.35	[-1.14; 0.44]	0.32
**VAS Scores (Morning after intervention)**			
Placebo treatment	-0.74	[-1.36; -0.12]	0.03*
Active treatment	-0.33	[-1.84; 1.19]	0.60

Analysis of change within the active and placebo treatment results for the owner completed ‘Helsinki chronic pain index (HCPI)’ and ‘visual analogue scale (VAS)’ is presented with the estimate of change from before treatment, lower and upper values of the confidence interval set at 95% and significance set as *P* < 0.05 marked with *. (*P* = level of significance)

## Discussion

The results of our study do not provide statistically significant evidence that one PEMFT treatment, using these specific treatment parameters, improves temporospatial gait variables or owner assessed pain in dogs with hip OA. Thus, in this study, the hypothesised treatment effect was not reached.

The only temporospatial measurement of gait to show a statistically significant change between active and placebo treatments from pre-treatment values was a shorter stride length of the treated hind limb (*P* = 0.03). Active and placebo treatments both demonstrated reduced reach post treatment with the reach of the active treatment being numerically clearly shorter than the placebo treatment, but this change was not statistically significant between active and placebo treatments (*P* = 0.19). The change in reach within the active treatment, however, was statistically significantly different compared to pre-treatment values (*P* = 0.04) which was not the case for the placebo treatment (*P* = 0.4). Clinically it is assumed, that range of motion and thus stride length would be limited by pain in a hind limb with hip OA, therefore the expectation was that these values would have increased after treatment. Although this reduction in reach was statistically significant, it amounted to approximately 2.11 cm in real terms so the overall effect on the dog’s gait and especially on its functionality is questionable. The fact that an effect, albeit different from the expected one, was shown in the active treatment results for reach and stride length may indicate that the dogs did feel a difference in the treated limb which may have felt strange to them, resulting in gait changes. Without repeating the gait analysis later that day, it is not possible to say whether this change was temporary until the dog adjusted to how the limb felt to them post treatment. Or perhaps the 20 min of lying down was a long enough time to result in OA related “stiffness after rest” response. Another explanation could relate to the incidence of bilateral disease within the sample. Six out of the eight dogs had bilateral disease and were required to lie on their opposite side for treatment application. It is possible that any treatment effect was negated by having the dog lie on their other side which may have caused residual pain or stiffness in what was considered on orthopaedic assessment to be their least painful side. Following treatment application, the dog may then have attempted to reduce weight bearing on the non-treated side which would result in shorter reach and stride length on the contralateral (treated) side [3]. Although a soft, supportive mat was used for dogs to lie on during the treatment, perhaps an even softer surface was required. If the PEMFT application had caused a detrimental effect on the painful joint, it would be expected that values of total pressure and stance time would show more change due to the dog wanting to offload weight from a painful limb [3]. Moreover, the owner assessment of their dog’s pain should have yielded some response, which it did not. The above hypotheses do not explain why the change in reach and stride length was significantly shorter within the active treatment results, but not the placebo treatment. One explanation could be linked to the behavioural observations made by the blinded clinician carrying out the treatment, who noted that some subjects lay much more still, relaxed, and settled than others during the treatment application. It was this observation that led the blinded clinician to correctly identify which applicator pad was which at the end of the study. The subjects receiving the true treatment application lay more still for the required 15 min. As a result, it is possible that these subjects were initially stiffer in the period when gait analysis was repeated.

The analysis of change in step length from pre-treatment values (Table 2), while not statistically significant between active and placebo treatments (*P* = 0.06) is worthy of note. The estimate within the active treatment, as seen in Table 3, is a negative value (-4.83) but positive for the placebo treatment (1.27) with a large confidence interval. The large confidence interval could be due to the number of subjects within the study and perhaps the sample was not big enough. With smaller sample sizes, it is possible for one subject with vastly different values, to skew the overall results. The fact that the change in step length between the groups (*P* = 0.06) is so close to the chosen significance level of *P* < 0.05 correlates with the result for stride length which was statistically significant (*P* = 0.03). Stride length is determined by length of steps, so it follows that the results for these two gait parameters are closely linked.

Our results did not show statistically significant immediate or short-term improvements in the selected outcomes with one application of PEMFT. Albeit not assessing immediate effect, the results of previous studies conflict ours, by demonstrating positive clinical effects in dogs with OA following PEMFT application [31, 35]. It is important to note, however, that our study used different PEMFT treatment parameters and outcome measures than previous studies. The mechanism of action of PEMFT is complex and the clinician has a choice of treatment parameters depending on the primary desired effect. For this study, the settings chosen (200 Hz, constant pulse, 15 min) targeted downregulation of pain pathways to produce an analgesic effect [39]. It is possible that selecting a lower frequency of 50 Hz to promote vasodilatation may relieve possible chronic swelling around the joint and demonstrate a different effect on signs of pain and lameness [20, 40]. In our study there was no statistically significant difference in HCPI or VAS scores between the treatment and placebo groups. The only statistically significant result for the owner assessed outcome measures was an improvement within the placebo group for the next morning score compared with pre-treatment VAS score. There are various possible explanations for our negative results which conflict with previously published research demonstrating positive clinical effects and improvement in pain score in dogs with OA following PEMFT application. Previous studies have relied on a variety of outcome measures, often subjective, not always using validated scales and demonstrating a lack of blinding of participants and researchers [31, 35]. Our study was randomised, controlled, double blinded and used carefully selected subjective and objective outcome measures shown to be valid and reliable [6, 7, 41]. In spite of this, it may be that the measures were not sensitive enough to detect very subtle changes in the subjects’ presentation following PEMFT treatment. For example, we employed the gold standard method of pressure sensitive walkway for kinematic gait analysis to determine the effect of PEMFT on lameness [41]. However, a recent study queried the sensitivity of pressure sensitive walkway as a diagnostic test for abnormal gait patterns caused by OA when the lameness was mild to moderate, as were the dogs in this study [42]. Another study suggested therapeutic responses were less likely to be identified if dogs were classed as mildly lame due to OA rather than more severe [43].

Validated and reliable outcome measures minimise bias, but unconscious selection bias may remain as owners may be more likely to score their dog more favourably following intervention, whether placebo or not [44]. The mean initial scores for both the HCPI and VAS were very low but with considerable variation between subjects as shown in the large SD (Tables 4 and 5). With low baseline scores, it may be difficult to detect any significant changes in a small study sample with wide variation in the datasets. The HCPI is not validated to be used as frequently as it was used in this study. It is possible that it is not sensitive enough or responsive enough to detect possible minute changes related to the intervention, when used with such short time intervals as we did, which may have affected our results from this part of the study. The lameness grading on orthopaedic examination by the specialist orthopaedic veterinarian for the sample was also low. The study cohort was symptomatically low to begin with, which perhaps makes it less likely that subtle effects with one treatment application would be recognised as there is little room for improvement. For the subjective and objective outcome measures used in this study, a larger, more symptomatic cohort with unilateral disease might have been more suited to detection of change in gait pattern and pain.

An unexpected result was the significant sequence effect of the crossover design on the VAS score. Subjects receiving active treatment first then placebo treatment, had a decrease in VAS score in the evening of the treatment day (*P* = 0.02) and the next morning (*P* = 0.06) which was not present for those subjects receiving placebo first and active treatment second. This is a recognised phenomenon but difficult to explain in this case. Carry-over effect can be a possible explanation but the two-week washout period in our study design and lack of demonstrable treatment effect makes this unlikely. A more likely explanation in this case is random chance occurrence due to small sample size. With only four dogs in each sequence, it is more likely that the subjects whose pain is decreasing are from the same group, giving the appearance of sequence effect. Within the placebo treatment results, there was a significant change in VAS measurement from pre-treatment score and next morning score (*P* = 0.03) as shown in Table 5.

This study was interested in the immediate effects on signs of pain and lameness assessed using owner reported pain scales and temporospatial gait analysis following only one application of PEMFT treatment. Investigating the effect of multiple treatments over a longer time period would be sensible as expecting immediate effects in such a chronic disease may be overly ambitious. We chose to study immediate effects of a single treatment based on observations of how this treatment modality is commonly used in clinical practice to help guide clinicians. Further research into the clinical benefits and optimal use of PEMFT within veterinary medicine is warranted. Direction of future study using similar methodology could include replicating current study with a larger sample and more severe signs of pain and lameness. Use of the ‘Canine OsteoArthritis Staging Tool (COAST)’ could be employed in future studies to grade sample subjects into subgroups of early, middle and late-stage OA [45]. It would be beneficial to study the effect of several PEMFT treatments or the effect of changing the parameters of the PEMFT machine to determine if that affects the outcome measures.

## Conclusions

The clinical impact of the current study suggests that one treatment application using these specific parameters does not provide immediate, measurable improvement in owner assessed pain levels or temporospatial performance in dogs with hip OA.

## Data Availability

Due to clinical nature of the data and study specific participant agreement and data management plan, the datasets will not be made available for external requests. All analysed data is available in the manuscript.

## Declarations

### Prior publication

Data have not been published previously.
